# The Fatty Acid Species and Quantity Consumed by the Breastfed Infant Are Important for Growth and Development

**DOI:** 10.3390/nu13114183

**Published:** 2021-11-22

**Authors:** Alexandra D. George, Melvin C. L. Gay, Mary E. Wlodek, Kevin Murray, Donna T. Geddes

**Affiliations:** 1 School of Molecular Sciences, The University of Western Australia, Crawley 6009, Australia; melvin.gay@uwa.edu.au (M.C.L.G.); m.wlodek@unimelb.edu.au (M.E.W.); donna.geddes@uwa.edu.au (D.T.G.); 2 Metabolomics Laboratory, Baker Heart and Diabetes Institute, Melbourne 3004, Australia; 3 Department of Obstetrics and Gynaecology, Faculty of Medicine, Dentistry and Health Sciences, The University of Melbourne, Melbourne 3010, Australia; 4 School of Population and Global Health, The University of Western Australia, Nedlands 6009, Australia; kevin.murray@uwa.edu.au

**Keywords:** infant nutrition, breastfeeding, lipidomics

## Abstract

The fatty acids (FAs) of human milk (HM) are the building blocks of the HM lipidome, contributing to infant health and development; however, this has not been comprehensively characterised with respect to infant intake. Eighteen Western Australian mother–infant dyads provided monthly longitudinal HM samples during six months of exclusive breastfeeding. Monthly anthropometric measurements, health data and basic maternal food frequency data were also collected. At three months, infant 24 h milk intake and total lipid intake were measured. The FA profile was analysed using gas chromatography–mass spectrometry. Linear regression and Pearson’s correlation were used to identify associations between HM FA composition, HM FA intake, maternal characteristics and infant growth and developmental outcomes. Mean infant intake of total lipids was 29.7 ± 9.4 g/day. HM FA composition exhibited wide variation between dyads and throughout lactation. Infant intake of a number of FAs, including C15:0, C18:1, C18:2 and C20:3, was positively related to infant growth (all *p* < 0.001). There were no relationships detected between C22:5 and C20:5 and infant head circumference. Infant total lipid intake and the infant intake of many FAs play essential roles in infant growth and development. This study highlights the important relationships of many HM FAs not previously described, including C15:0 and C18:2 species. Infant outcomes should be considered in the context of intake in future HM studies.

## 1. Introduction 

The composition and role of fatty acids (FAs) in human milk (HM) has been investigated and described for over 60 years [1]. These FAs are the building blocks of the HM lipidome, forming triacylglycerides, representing approximately 98% of the lipidome, and other lipids such as phospholipids and gangliosides, which comprise approximately 2% of the lipidome [2]. Incorporation of FAs into the different lipid structures originates from either endogenous synthesis, or via maternal circulation originating from maternal diet or tissues stores [3]. The FAs within HM are well known to be influenced by maternal diet, for example, maternal fish intake is associated with docosahexaenoic acid (C22:6) abundance in HM [4]. Both long- and short-term dietary habits have been previously shown to influence maternal milk FA composition [5]. A number of other maternal factors have also been shown to influence the HM FA composition, including, but not limited to, BMI and genetic variants of FA desaturase (FADS) genes [6,7]. 

The FAs profile of HM displays wide variation between dyads, and over the years, some of the importance of HM FAs for the infant has been described and postulated [8]. In particular, there has been great research interest surrounding arachidonic acid (C20:4) and docosahexaenoic acid (C22:6), which appear to influence infant growth, brain growth and cognitive function [9]. While some previous studies have included large datasets to elucidate relationships between HM FAs and infant outcomes, infant daily FA intake has only been reported once [10]. Because infants consume milk of different compositions and in different volumes, varying from approximately 500 mL/day to 1000 mL/day, it is important to consider both the overall profile of the HM FAs and the intake of each HM component when exploring relationships with maternal impact or infant outcomes [11]. Volume consumed can be measured using either the test weighing or deuterium dilution method. To measure daily infant intake of HM components, feed volumes are combined with mean feed concentrations [12].

Despite the wide variation in both HM FA composition and HM intake, measurement of infant intake of specific lipid species is rare and is likely an important aspect of HM research. The aim of this study was to investigate infant fatty acid intake and interrogate the relationships between infant intake and infant growth and development from birth to six months.

## 2. Methods

### 2.1. Recruitment, Data Collection and Human Milk Sampling

Pregnant women who intended to exclusively breastfeed were recruited for a longitudinal human milk lipidomics study and provided background information including age, education, parity, and health. Mother–infant dyads were followed from birth to six months of exclusive breastfeeding. At birth, infant weight, length, head circumference, sex and delivery mode were also collected. Thirty mother–infant dyads were recruited; however, dyads were excluded from this study if supplementary feeding was initiated within six months, if there were any health or growth concerns, if a full sampling data set was not collected, or if there was inadequate sample volume to analyse all samples. This cohort details 18 mother–infant dyads and a total of 704 HM samples. This study was approved by The University of Western Australia Human Research Ethics Office, RA/4/20/4023, and all participants provided informed, written consent.

During exclusive breastfeeding, sampling time points were monthly (±2 days). On the sampling day, three pre-feed HM samples (≤2 mL) were collected manually by each participant, morning: 0600–0900 h, afternoon: 1300–1600 h and evening: 1900–2200 h, from the feeding breast. Pre-feed samples were collected due to frequent sampling, for ease of collection and consistency. In addition, a basic maternal food frequency questionnaire was completed, questioning intake of specific foods (oils, fish, meats, eggs, nuts, avocado and DHA-containing supplements) selected based on their FA composition, in both the 24 h prior to sample collection and for the preceding month. Each mother provided information via short questionnaires about infant health and achievement of developmental milestones. Infant weight using baby weigh scales with ±2 g accuracy (Medela Inc., McHenry, IL, USA), length using a measuring board (Seca, Chino, CA, USA) and head circumference, HC, using nonstretch tape measure were measured. Maternal height and weight, using electronic scales (Seca, Chino, CA, USA), were measured, and body mass index (BMI) was calculated as mass (kg)/height (m)^2^. To measure daily infant milk intake and estimate total daily lipid intake, at three months, 24 h test-weighing and milk sampling were carried out. Infants were weighed before and after each feed, and pre- and post-feed milk samples (≤2 mL) were collected [11]. All samples were stored by the mother at −20 °C and were transferred to −80 °C, within 24 h of collection, for storage. The sampling and data collection protocol is summarised in Supplementary Table S1.

### 2.2. Human Milk Fatty Acid Analysis

Human milk samples (200 µL) were prepared and analysed as fatty acid methyl esters (FAMEs), based on previously published methods [13]. Biphenyl solution (1.0 mL; 70 mM) was used as the internal standard (IS). The HM and IS mixture was saponified with 1.0 mL of 0.5 M sodium hydroxide in methanol and refluxed for 10 min at 70 °C. The mixture was derivatized with boron trifluoride solution (0.5 mL) and further refluxed for 3 min at 70 °C. Liquid–liquid extraction and salting out were carried out with heptane (1 mL) and saturated sodium chloride solution (1 mL), respectively. The solution was left to separate for 10 min, and the heptane layer was extracted. The extract (0.3 µL) was injected into the GC-MS (Trace1310 GC, coupled with a single quadrupole MS (ISQ LT; Thermo Fisher Scientific, San Jose, CA, USA)) with a Supelco SLB-IL 111 column (200 m × 0.25 mm × 0.2 µm; Sigma-Aldrich Co. LLC, St. Louis, MO, USA). The initial column temperature was 120 °C and was maintained for 13 min. The temperature was increased at 2 °C/min to 150 °C and maintained for 4 min. Then, it was increased at 1.6 °C/min to 190 °C and maintained for 5 min. Then, it was increased at 1.6 °C/min to 220 °C and maintained for 3 min. Finally, it was increased at 10 °C/min to 240 °C and for 3 min, with a total run time of 95 min. Helium (1.2 mL/min at constant flow) was used as the carrier gas. The mass range acquired was 45 to 500 amu, and the ion source temperature was set at 270 °C. Quality control (QC) samples (pooled and technical QC) were analysed every 20 samples. FAMEs were identified using the National Institute of Standards and Technology (NIST) library. Forty-six FAMEs with acceptable reproducibility were included for further statistical analysis (pooled and technical QC relative standard deviation <30% or <0.1% for very-low-abundance species).

### 2.3. Daily Milk, Total Lipid and Fatty Acid Intake 

The daily milk intake was calculated by summing the weighed intake volumes of each feed over a 24 h period. Based on previous research that infant milk intake does not change during exclusive breastfeeding, this volume was assumed to be static from months one to six [14]. The total lipid content of each HM sample was measured in triplicate (RSD ≤ 1%) using the creamatocrit method. The mean lipid percentage for each triplicate sample was converted to concentration (*g/L*) using the following equation [15]:

$$total\;lipid\;concentration\;\left(\frac{g}{L}\right)=\left(creamatocrit\left(\%\right)-0.59\right)/0.149$$

At three months lactation, the total daily lipid intake for each infant was calculated by summing the mean pre- and post-feed lipid content and feed volume, for each feed in the 24 h period. In months one, two, four, five and six, the average lipid content (three pre-feed samples) was multiplied by the measured daily milk intake (at three months). Each infant’s fatty acid intake per month was estimated using the average percent of each FA from each time point multiplied by the total daily lipid intake.

### 2.4. Statistical Analysis

Statistical analysis was carried out using Rstudio (Version 1.3.1093). Comparisons for monthly cohort growth characteristics (maternal weight and BMI, infant head circumference, length and weight measurements, raw and z scores, and infant BMI) were carried out using repeated-measures ANOVA. HC was used as a proxy for brain size and growth, which may be linked to infant development. Monthly responses to maternal basic food frequency questionnaires were entered as scores (‘0’ corresponded to never, ‘1’ corresponded to once per week, ‘2’ corresponded to between one and three times per week, ‘3’ corresponded to between four and six times per week, and ‘4’ corresponded to every day). For the 24 h prior to HM sample collection, responses were scored as either ‘no’ (1) or ‘yes’ (2). Daily, pre- and post-feed, and monthly FA composition was compared using repeated-measures ANOVA. Variation for FA percentages and infant intake was assessed using relative standard deviation (RSD). Pearson’s correlation identified relationships between maternal factors such as diet and FA proportions, and between infant intake and infant growth outcomes at each month. Heat maps were constructed to visualise relationships between infant intake and infant growth characteristics. In all cases, *p* < 0.05 was reported as significant due to small sample size; however, Benjamini–Hochberg correction was also carried out to reduce false discovery rate, to further interrogate relationships.

## 3. Results

### 3.1. Study Cohort

Eighteen healthy breastfeeding mothers of term (>37 weeks) infants, nine female and nine male, completed this study (Table 1).

The majority of the participating mothers had tertiary-level education (*n* = 17), and the remaining mother had completed high school education (*n* = 1). Fifteen of the infants were delivered vaginally, and three were born by caesarean section. Dyads were healthy throughout the study period, with the exception of occasional cold-like illnesses. Maternal weight and BMI decreased significantly from months one to six of lactation. Infants were growing appropriately on their respective weight-for-length WHO growth curves. Infant HC, weight, length, HC z-score and BMI all increased significantly from birth to six months exclusive breastfeeding (Table 2).

### 3.2. Maternal Diet

Based on the basic food frequency questionnaire, all mothers ate relatively similar diets and these remained consistent throughout lactation. No mothers were vegetarian or vegan, and all consumed fish regularly (one to three times per week) and/or regular fish oil supplements throughout pregnancy and lactation. No mothers reported having any food allergies. Dietary differences were present for egg, nut and avocado frequency, ranging from never to daily throughout lactation. For the other foods included in the questionnaire, no large differences were noted and thus they were not subsequently investigated (Supplementary Table S2).

### 3.3. Fatty Acid Composition of Human Milk 

Forty-six distinct FAs were separated and quantified in all human milk samples, ranging from hexaenoic acid (C6:0) to tetracosanoic acid (C24:1) (FA details and proportions can be found in Supplementary Table S3). In addition to linoleic acid (C18:2, cis-9, cis-12 octadecadienic acid), three other C18:2 isomers, trans-9, trans-12 octadecadienic acid, cis-9, trans-12 octadecadienic acid and trans-9, cis-12 octadecadienic acid, were identified. Four other species were identified as CLA isomers (C18:2), based on library identification as C18:2 (cis-9, cis-12 octadecadienic acid) but with longer retention times. 

No significant differences in FAs were observed between the three-month pre-feed and post-feed samples (*p* > 0.05), such that using either did not affect overall FA percentages, despite a significant increase in total lipid concentration between pre- and post-feed samples (mean difference: 28.9 ± 19.8 g/L; 95% CI: 26.1–31.7; *p* < 0.001). The FAs present in the highest amounts were oleic acid (C18:1, cis-9-octadecanoic acid, mean percent of total: 37.5%), palmitic acid (C16:0, mean percent of total: 21.7%), linoleic acid (C18:2, 11.6%), steric acid (C18:0, mean percent: 7.5%), and myristic acid (C14:0, 5.4%), comprising over 80% of the total FA composition. 

No obvious monthly trends were identified for the relative percentage of each FA. The variation (RSD) for each FA between women ranged from 0% (all samples contained 0.01% cis-10-pentadecanoate, C15:1) to 191% (for cis-11,14,17-eicasatrienioc acid, the relative percentage ranged from 0.01% to 0.33%) (Supplementary Table S3).

### 3.4. Maternal Diet and Human Milk Fatty Acid Composition

Relationships were identified between maternal food frequencies and specific FA composition. Due to the low dietary variation, only relationships with eggs, nuts and avocados were analysed, as the intake frequency scores were distributed from ‘never’ to ‘daily’ for these foods. Maternal food intake was significantly related to the abundance of a number of HM FAs, including eicasatrienoic acid (C20:3*n*-6), CLA3 (C18:2), γ-linolenic acid (C18:3*n*-6) and linoleic acid (C18:2*n*-6) (*p* < 0.001; Supplementary Table S4). Maternal diet for the foods of interest in the 24 hours preceding HM sample collection did not influence any FA proportions. In this cohort, HM FA composition was not correlated with any other maternal factors (age, BMI, weight, weight change over lactation, parity, or education).

### 3.5. Relationships between Maternal Characteristics, Infant Intake, and Infant Growth Characteristics

The mean infant total lipid intake was 29.7 ± 9.4 g/day (range: 11.3–49.1 g/day). Infant milk intake had positive correlations with infant head circumference (*p* = 0.013) and infant weight (*p* = 0.029). No maternal factors (such as weight, BMI or age) were related to infant milk intake or infant outcomes, although the cohort was relatively homogenous. 

**Figure 1:**
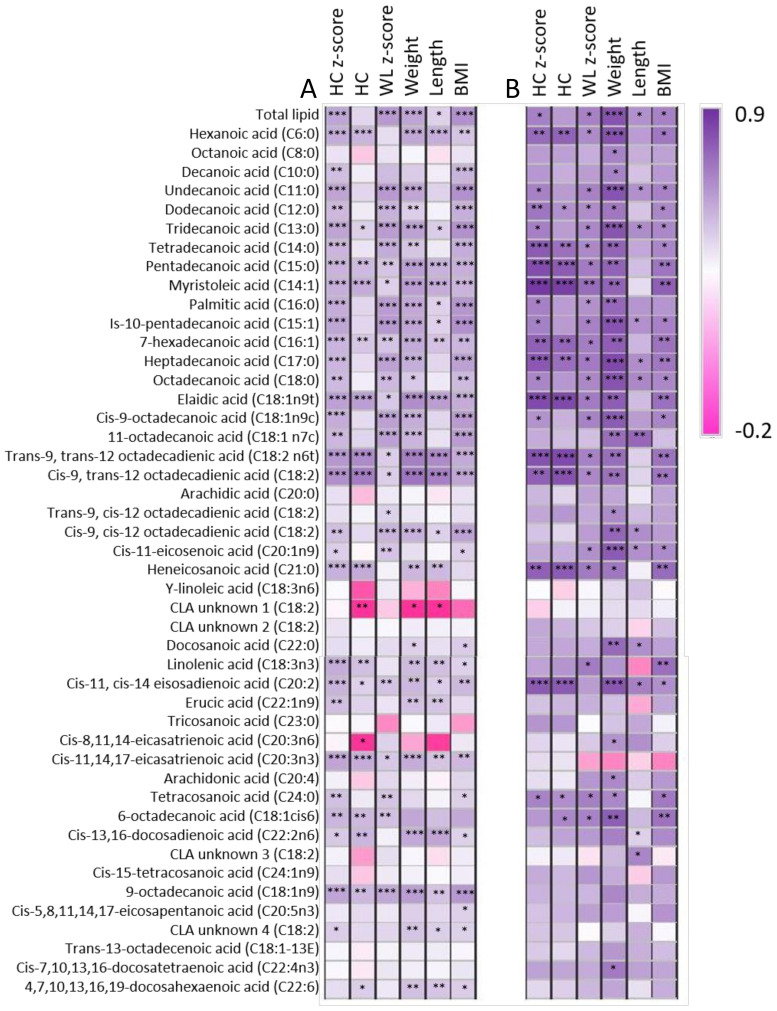
Relationships between infant lipid intake and infant growth characteristics. (**A**) Monthly intake and growth characteristics, (**B**) Mean six-month intake and six-month growth characteristics. Dark purple represents strongest positive correlation, and dark pink represents strongest negative correlation. * Indicates *p* < 0.05, ** indicates *p* < 0.01, *** indicates *p* < 0.001 (*p* < 0.001 is significant after accounting for multiple comparisons).

Daily infant intake of the 46 different FAs displayed wide variation between infants (Supplementary Table S5). The total intake of *n*-3 and *n*-6 FAs did not differ significantly through lactation (*p* > 0.05); however, the AA:DHA ratio in month two was above 15 for six of the dyads (full range: 0.9–25.8). Similarly, the mean *n*-6:*n*-3 ratio in month two was higher than that of the other months, due to approximately half the dyads having high ratios (full range: 15.5–331.6). As total *n*-3, total *n*-6 and ratios of *n*-6:*n*-3 and AA:DHA are frequently assessed, we also analysed these with respect to infant growth characteristics. There were no associations identified between the total *n*-3 intake, total *n*-6 intake, the *n*-6:*n*-3 ratio or the AA:DHA ratio and infant growth characteristics. Additionally, several significant positive relationships were identified between infant FA intake and infant growth characteristics, particularly for the medium-chained FAs, many of which remained significant after Benjamini–Hochberg adjustment (Figure 1A). Strong positive relationships were demonstrated between infant intake of a number of FAs and infant growth characteristics (excluding WL z-score). Among these were trans-9, trans-12 octadecadienic acid (intake range: 0.00–0.12 mg/day) and cis-9, trans-12 octadecadienic acid (intake range: 0.84–7.65 mg/day). The few weak negative relationships were rendered insignificant after Benjamini–Hochberg adjustment. The strongest relationships were identified between mean intake over months one to six of exclusive breastfeeding and infant growth characteristics at six months. In particular, mean infant intake of most medium-chained FAs demonstrated strong positive relationships with infant weight at six months (*p* < 0.001, Figure 1B). Infant head circumference (both raw and z-score) had positive relationships with the intake of C15:0, C14:1, C18:1, C18:2 and C20:2. Similar positive relationships were identified between average intake of all medium-chained FAs and many long-chained FA through lactation and infant weight at 12 months (*p* < 0.001). No significant relationships between infant intake of DHA and infant characteristics at any month were identified; however, because DHA is frequently measured in relation to infant brain development, we compared infant HC and HC z-score with percent DHA and both had weak positive relationships.

## 4. Discussion 

Human milk fatty acids have been a research area of high interest due to their proposed involvement in infant growth and development, yet we still do not have a comprehensive knowledge and understanding of this. In this study, we report the FA intake for a cohort of healthy exclusively breastfeeding infants from birth to six months and identify many significant relationships between infant intake of FA and infant growth characteristics.

The 46 FAs identified in this study ranged from C6:0 to C24:1 and included many unsaturated and odd-chain FAs such as C11:0, C14:1, C15:1 and eight different C18:2 species, not routinely reported. Given the apparent importance of low-abundance FAs, such as DHA and AA, it is important that as many species be incorporated into analysis as possible. HM FA percentages differ greatly between studies due to the different total numbers of FAs being reported, but previous studies have typically reported a lower number of FA species, likely due to co-elution of various FAs, elution below detection limits, or FA exclusion due to poor reproducibility. Reported FA differences are also a result of varying maternal diets across different populations, an important consideration, as many FAs such as C18:2, are only derived from food sources [16]. Whilst the majority of saturated FAs up to C16:0 are synthesised in the mammary gland from acetyl-CoA and malonyl-CoA, using synthetase and thioesterase enzymes, wide percent variation still existed between dyads, including for C12:0 (0.2–8.7% total FA), C14:0 (1.8–11.4% total FA) and C16:0 (13.1–27.8% total FA), all consistent with previous studies [6,7]. These differences may reflect maternal genetic variation in the FA synthetase cycle, whereby metabolism and homeostasis are regulated differently at certain reactions along the synthetic pathway. Other enzymes have been shown to differ between women, including the enzyme that desaturates FAs by removing hydrogen to create a double bond, FADS [6,17].

Of all the potential mechanisms through which the lactating mother may influence HM FA composition, maternal dietary influence is an important consideration as a potential intervention point to alter milk composition for optimal infant outcomes. Interestingly, our study cohort, consisting of healthy, well-educated women, consumed relatively similar diets (based on the basic food frequency questionnaire), yet there was wide variation in HM FA composition and concentration (RSD > 100 for many species) between women. As such, we found significant relationships between maternal egg, nut and avocado intake frequency and HM FA composition (Supplementary Table S4). In Australia, the majority of the population does not meet the recommended DHA intake; however, all the women in this cohort reported regular fish and DHA-containing supplement intake [18]. This conflicts with the high variation observed for the relative abundance of HM DHA, ranging from 0.01–0.67% (mean ± SD: 0.20 ± 0.14%), although we cannot quantify maternal DHA intake in this study. This variation was in accordance with previous studies including Canadian (0.18 ± 0.12%), United States (0.26 ± 0.13%) and Australian cohorts (week 6: 0.26 ± 0.13%; week 16: 0.21 ± 0.13%, week 30: 0.19 ± 0.10%). In contrast, the mean was higher than the 0.13% reported in another United States cohort, and considerably lower than other Western Australian (0.36 ± 0.3%) and Bolivian cohorts (0.62%) [6,7,9,19,20]. The evidence that dietary DHA directly influences HM DHA proportions remains inconclusive as multiple studies have reported no, or weak, relationships between maternal DHA intake and HM DHA [5,21,22]. Observational studies, like our own, have previously identified relationships using food frequency questionnaires, but higher DHA content may in fact reflect an overall healthier, balanced diet. One fish oil intervention study demonstrated very high DHA proportions in the HM of women supplemented during pregnancy and lactation (decreasing from day 4 to 30 post-partum: from 1.8% to 1.4%) compared to women supplemented only in pregnancy (decreasing from 1.2% to 0.6%) compared to women not supplemented at all (decreasing from 0.9% to 0.5%) [23]. Similarly, HM DHA was significantly higher in women who were supplementing with fish oil in pregnancy (1.15 ± 0.5% compared to 0.5 ± 0.2%) [24]. Collectively, these studies led us to expect that the HM DHA proportion would be considerably higher in our cohort, but it appears other factors, particularly body stores, may be regulating HM DHA composition. This is supported by a study that supplemented lactating women with carbon-13-labelled DHA and demonstrated that only approximately 20% of dietary DHA contributes to the HM DHA composition [5]. The proportion of HM DHA, and other FAs, however, does not represent the quantity consumed by the infant as they consume vastly different milk volumes (473–946 mL/day in this study); thus, we calculated infant FA intake, which enabled elucidation of relationships with infant growth [25].

Total infant lipid intake ranged from 11.3 to 49.1 g/day and was positively related to infant HC z-score, weight and weight-for-length z-score, and BMI (all *p* < 0.001). This result is not entirely unexpected, as HM lipids provide the majority of the infant calorie intake [26]. Intake of many, but not all, lipids was also related to infant growth characteristics through the period of exclusive breastfeeding, therefore indicating both total and individual lipid intake are important for infant growth (Figure 1). Intake of medium-chain FAs, many of which are endogenously synthesised, had positive relationships with infant growth characteristics, for which there may be distinct maternal genetic differences contributing to this. This included odd-chained FAs C11:0, C13:0 and C15:0, as well as monounsaturated species like C14:1, C15:1 and C16:1. Many odd-chain FAs appear to have health benefits in adults, whereby higher intake of odd-chained FAs is associated with lowered risk of inflammation, cardiovascular disease and type 2 diabetes. Breastfed infants are at lower risk of all of these compared to formula-fed infants [27]. The pathway through which C15:0 acts is as an agonist of peroxisome proliferator-activated receptors (PPAR), which regulate metabolism and inflammation in response to fat. PPAR gamma has previously been implicated in infant health, where PPAR gamma polymorphisms were identified in association with early childhood adiposity [28]. It is therefore conceivable that C15:0 may be contributing positively to infant growth. The high-abundance HM FA palmitic acid has previously been implicated in immunity, increasing in HM when the infant is unwell, although this has not been well characterised [7]. In addition, the observed positive relationships between palmitic acid intake and infant HC, weight, weight-for-length z-score and BMI may be mediated through support of the infant immune system.

After Benjamini–Hochberg adjustment, relationships with HC and infant intake of C18:0, C18:1 and C18:2 species, C21:0, C18:3*n*-3, C20:2 and C20:3 remained significant. Interestingly we did not observe relationships with C22:6 (DHA) and C20:5 (eicosapentaenoic acid) FAs but did identify a positive relationship with intake of C18:3, another omega-3 FA. Omega three (*n*-3) and omega six (*n*-6) species have previously been linked to infant visual acuity and neurodevelopmental outcomes, as integral structural components of neural cell membranes. The visual acuity of infants fed HM, fish oil-supplemented formula and non-enriched formula has previously been compared, with researchers finding infant erythrocyte DHA was maintained in the HM and fish oil-enriched formula groups, but not in the standard formula group, and positively correlated with infant visual acuity scores [29]. These, and the *n*-6:*n*-3 ratios, are of particular interest because more nutrient deficient modern diets appear to be altering the *n*-6 and *n*-3 proportions and *n*-6:*n*-3 ratios have been implicated in infant fat mass accumulation [30]. However, our ratios ranging from 26.79 ± 7.33 to 84.50 ± 83.29 were considerably higher than those of previous reports, and we measured much lower total *n*-3 than total *n*-6. Intake of *n*-6 has previously been shown to alter the *n*-3 levels, which may be the case in our cohort. Another ratio of frequent interest is AA:DHA, although its importance is unclear [1]. The AA:DHA range we reported aligned with that of other studies, although ratios were highly variable through lactation, highest in month two (8.97 ± 9.37) and lowest in month six (0.77 ± 1.01). This also suggests endogenous regulation, as maternal diet remained consistent through lactation. Studies have frequently linked the percent of DHA in HM to infant HC or neural development, for which we observed a weak positive relationship with HC; however, when intake was considered, there were no significant relationships between HC z-score and DHA intake, and the weak positive relationship between HC and DHA intake (*p* < 0.05) was rendered insignificant after correction for multiple comparisons. This finding provides some evidence as to why no conclusive evidence exists for maternal DHA intake and infant neurodevelopmental outcomes. It also emphasises that relationships with infant intake can differ greatly compared to concentrations when considering infant outcomes. Where infant outcomes are concerned, a key factor in understanding the role of these FAs will be to measure infant intake in future studies, by studying exclusively breastfeeding cohorts and recording other food sources that may have a confounding effect [25].

Despite collection of 704 HM samples and longitudinal data for six months from exclusively breastfeeding dyads, the sample size (*n* = 18) and homogeneity of this cohort are the main limitations within the study. Additionally, the basic maternal food frequency questionnaire did not allow precise and quantitative assessment of the contribution that maternal diet has on HM composition. Future studies should focus on these factors, collecting data and samples from larger and more comprehensive cohorts, and using more in-depth methodology to assess maternal diet. Regardless, many of the relationships we identified between infant growth and FA intake remained after Benjamini–Hochberg adjustment. These findings provide the foundation for the development of future studies.

## 5. Conclusions

With >700 samples and a cohort of 18 mother–infant dyads who exclusively breastfed for six months, we were able to comprehensively assess infant intake of HM FAs. Many significant relationships were identified between infant intake and infant growth, particularly infant weight, reinforcing the importance of HM FAs. Further, longitudinal studies and intake measurements are essential to determine maternal influence on HM FAs and the mechanisms through which HM FAs contribute to infant growth and health.

## Figures and Tables

**Table 1:** Cohort characteristics of 18 mother–infant dyads.

	Cohort Values (Mean ± SD (Range))
Maternal age at delivery (years)	32.0 ± 3.3 (26.0–37.1)
Parity (*n*)	2.0 ± 1.7 (1.0–3.0)
Gestational age (weeks)	40.1 ± 1.6 (37.7–41.7)
24 h milk intake (mL/day)	740.8 ± 150.2 (473.0–946.0)

**Table 2:** Mean maternal and infant growth characteristics from months one to six exclusive breastfeeding.

	Birth	1 Month	2 Months	3 Months	4 Months	5 Months	6 Months	*p*-Value
Maternal weight (kg)		78.9 ± 18.1	78.5 ± 18.6	77.7 ± 17.8	77.1 ± 17.5	76.2 ± 16.9	76.5 ± 17.2	0.0147
Maternal BMI (kg/m^2^)		28.4 ± 6.2	28.2 ± 6.4	27.9 ± 6.3	27.7 ± 6.2	27.4 ± 6.0	27.5 ± 6.1	0.0108
Infant weight (kg)	3.9 ± 0.4	4.8 ± 0.6	5.6 ± 0.7	6.2 ± 0.8	6.8 ± 0.9	7.4 ± 1.0	7.9 ± 0.9	<0.0001
Infant length (cm)	52.3 ± 1.4	55.4 ± 2.1	58.7 ± 2.2	61.3 ± 2.2	63.7 ± 2.4	65.4 ± 2.4	67.0 ± 2.4	<0.0001
Infant w-l z score	0.0 ± 0.9	0.4 ± 0.9	0.1 ± 1.1	0.0 ± 1.1	0.0 ± 1.1	0.3 ± 1.1	0.4 ± 1.1	0.107
Infant HC (cm)	36.1 ± 0.7	38.2 ± 1.0	40.0 ± 1.1	41.4 ± 0.9	42.5 ± 1.3	43.5 ± 1.2	44.5 ± 1.4	<0.0001
Infant HC z score	−0.2 ± 0.4	−0.2 ± 0.6	0.0 ± 0.7	0.2 ± 0.6	0.5 ± 0.7	0.7 ± 0.7	0.9 ± 0.8	<0.0001
Infant BMI (kg/m^2^)	14.1 ± 1.1	15.5 ± 1.1	16.2 ± 1.5	16.5 ± 1.5	16.8 ± 1.7	17.3 ± 1.9	17.5 ± 1.8	<0.0001

Data are mean ± standard deviation; HC: head circumference; BMI: body mass index; maternal characteristics were not collected at infant birth.

## Data Availability

Study data is available to researchers upon reasonable request.

## Supplementary Materials

The following are available online at https://www.mdpi.com/article/10.3390/nu13114183/s1. Table S1: Sampling, measurement and data collection timing protocol, Table S2: Maternal food frequency in the month prior to sample collection, Table S3: Fatty acids identified, naming conventions and percent of total (46 fatty acids) throughout lactation, Table S4: Significant relationships between human milk fatty acids and maternal intake frequency for eggs, nuts and avocado, Table S5: Infant fatty acid intake (mg/day) for exclusively breastfeeding infants from months one to six of lactation.
