# Supplementary material for: The Fatty Acid Species and Quantity Consumed by the Breastfed Infant Are Important for Growth and Development
Source: Nutrients. 2021 Nov 22;13(11):4183. doi: 10.3390/nu13114183 (PMC8621480; doi:10.3390/nu13114183)
Supplement: Supplementary file 1 [file nutrients-13-04183-s001.zip › nutrients-1469187-SI.pdf]

The fatty acid species and quantity consumed by the breastfed infant are important for growth and development

Supplementary material

Supplementary Table S1. Sampling, measurement and data collection timing protocol Page 1

Supplementary Table S2. Maternal food frequency in the month prior to sample collection Page 2

Supplementary Table S3. Fatty acids identified, naming conventions and percent of total (46 fatty acids) throughout lactation Page 3

Supplementary Table S4. Significant relationships between human milk fatty acids and maternal intake frequency for eggs, nuts and avocado Page 6

Supplementary Table S5. Infant fatty acid intake (mg/day) for exclusively breastfeeding infants from months one to six of lactation Page 7

Supplementary Table S1. Sampling, measurement and data collection protocol

Collection	Month						
	0	1	2	3	4	5	6
**Infant weight, length and head circumference**	x	x	x	x	x	x	x
**Maternal weight and height**		x	x	x	x	x	x
**Maternal food frequency questionnaire**		x	x	x	x	x	x
**Pre-feed milk samples, from feeding breast (morning, afternoon and evening)**		x	x	x	x	x	x
**Infant milk intake**				x			
**Pre- and post-feed milk samples for 24 hours**				x			
**Infant growth, health and development questionnaire**	x	x	x	x	x	x	x

Supplementary Table S2. Maternal food frequency in the month prior to sample collection. Monthly frequency numbers were allocated as: never (0), ≤once per week (1), between one and three times per week (2), between four and six times per week (3) and every day (4). Results are presented as mean±SD

	OLIVE/CANOLA OIL	COCONUT OIL	FISH	RED MEAT/CHICKEN	EGGS	NUTS	AVOCADO	DHA-CONTAINING SUPPLEMENTS
**Month 1**	3.6±1.3	1.6±2.0	1.3±0.7	3.4±0.7	2.5±1.2	2.4±1.5	2.2±1.2	2.5±2.0
**Month 2**	3.6±1.3	1.7±2.0	1.3±0.7	3.3±0.7	2.3±1.2	2.3±1.3	2.3±1.3	2.3±2.0
**Month 3**	3.6±1.3	1.8±2.1	1.4±0.7	3.4±0.7	2.4±1.1	2.1±1.4	2.1±1.2	2.4±1.9
**Month 4**	3.6±1.3	1.8±2.1	1.4±0.7	3.4±0.7	2.3±1.1	1.9±1.3	2.3±1.2	2.1±2.0
**Month 5**	3.6±1.3	1.6±2.0	1.3±0.7	3.4±0.7	2.5±1.2	2±1.3	2.2±1.2	2.5±2.0
**Month 6**	3.6±1.3	1.6±2.0	1.3±0.7	3.4±0.7	2.6±1.2	2.1±1.4	2.2±1.3	2.3±2.0

Supplementary Table S3. Fatty acids identified, naming conventions and percent of total (46 fatty acids) throughout lactation. Results are listed monthly as mean±SD and median (Q1, Q3).

**Formula**	**Fame identity**	**Month 1**	**Month 2**	**Month 3**	**Month 4**	**Month 5**	**Month 6**	**RSD**
C6:0	Methyl hexanoate	0.012±0.007<br>0.010 (0.010,0.010)	0.013±0.006<br>0.010 (0.010,0.010)	0.011±0.004<br>0.010 (0.010,0.010)	0.013±0.005<br>0.015 (0.011,0.021)	0.017±0.015<br>0.01 (0.01,0.013)	0.015±0.012<br>0.01 (0.01,0.019)	67.25
C8:0	Methyl octanoate	0.161±0.104<br>0.110 (0.014,0.199)	0.133±0.084<br>0.227 (0.107,0.289)	0.138±0.124<br>0.139 (0.081,0.265)	0.144±0.102<br>0.147 (0.096,0.206)	0.146±0.100<br>0.108 (0.071,0.148)	0.151±0.117<br>0.080 (0.036,0.158)	71.35
C10:0	Methyl decanoate	1.673±0.524<br>1.492 (1.324,1.597)	1.533±0.251<br>1.670 (1.541,1.949)	1.603±0.452<br>1.897 (1.483,2.205)	1.653±0.374<br>1.679 (1.250,1.905)	1.605±0.376<br>1.334 (1.235,1.443)	1.615±0.572<br>1.493 (1.284,1.660)	26.65
C11:0	Methyl undecanoate	0.010±0.000<br>0.010 (0.010,0.010)	0.010±0.000<br>0.010 (0.010,0.010)	0.010±0.000<br>0.010 (0.010,0.010)	0.010±0 0.010<br>(0.010,0.010)	0.01±0.00<br>0.010 (0.01,0.01)	0.01±0<br>0.010 (0.01,0.01)	0.00
C12:0	Methyl dodecanoate	4.678±1.719<br>4.507 (3.959,5.888)	4.17±1.138<br>4.619 (3.437,5.109)	4.294±1.533<br>4.599 (3.976,5.999)	4.657±1.325<br>5.762 (3.327,6.577)	4.482±1.611<br>3.848 (3.187,4.95)	5.26±1.661<br>4.292 (3.831,4.955)	33.06
C13:0	Methyl tridecanoate	0.010±0.001<br>0.010 (0.010,0.010)	0.010±0.001<br>0.010 (0.010,0.010)	0.010±0.001<br>0.010 (0.010,0.010)	0.010±0.000<br>0.010 (0.010,0.010)	0.01±0<br>0.01 (0.01,0.01)	0.001±0.000<br>0.010 (0.01,0.01)	9.23
C14:0	Methyl tetradecanoate	5.619±2.117<br>5.939 (5.375,7.139)	5.266±1.648<br>4.868 (4.175,5.432)	4.916±1.47<br>4.467 (3.241,6.477)	5.198±1.420<br>5.611 (2.931,6.267)	5.621±1.846<br>5.104 (4.724,6.607)	5.895±1.663<br>5.541 (4.885,6.795)	31.4
C15:0	Methyl pentadecanoate	0.356±0.179<br>0.325 (0.279,0.425)	0.369±0.156<br>0.230 (0.216,0.326)	0.352±0.122<br>0.213 (0.176,0.44)	0.332±0.136<br>0.238 (0.157,0.443)	0.343±0.15<br>0.481 (0.440,0.545)	0.281±0.14<br>0.406 (0.333,0.478)	43.62
C14:1	Methyl myristoleate	0.274±0.172<br>0.208 (0.118,0.262)	0.236±0.123<br>0.163 (0.128,0.203)	0.236±0.107<br>0.113 (0.073,0.333)	0.223±0.104<br>0.162 (0.078,0.372)	0.26±0.177<br>0.336 (0.293,0.369)	0.208±0.131<br>0.324 (0.254,0.355)	57.27
C16:0	Methyl palmitate	21.801±3.530<br>22.278 (21.705,23.163)	22.454±2.353<br>21.102 (20.195,22.662)	21.669±2.566<br>23.428 (19.723,24.015)	21.451±2.669<br>17.254 (15.977,19.906)	21.672±3.387<br>24.088 (22.515,24.913)	21.075±3.05<br>22.35 (21.834,24.063)	13.45
C15:1	Methyl cis-10-pentadecenoate	0.010±0.000<br>0.010 (0.010,0.010)	0.010±0.000<br>0.010 (0.010,0.010)	0.010±0.000<br>0.010 (0.010,0.010)	0.010±0.000<br>0.010 (0.010,0.010)	0.01±0.000<br>0.010 (0.010,0.010)	0.01±0 0.01 (0.01,0.01)	0.00
C16:1	Methyl 7-Hexadecenoate	0.478±0.136<br>0.406 (0.368,0.525)	0.421±0.129<br>0.369 (0.268,0.39)	0.417±0.107<br>0.328 (0.229,0.376)	0.492±0.283<br>0.458 (0.433,0.510)	0.453±0.114<br>0.506 (0.469,0.602)	0.369±0.132<br>0.501 (0.479,0.525)	37.29
C17:0	Methyl heptadecanoate	2.82±0.668<br>2.927 (2.698,3.316)	2.786±0.425<br>2.61 (2.353,2.751)	2.825±0.626<br>2.366 (2.244,3.019)	2.774±0.587<br>2.347 (1.983,3.017)	2.778±0.587<br>2.636 (2.549,3.084)	2.64±0.619<br>2.988 (2.735,3.35)	20.92
C18:0	Methyl octadecoanate	7.436±1.925<br>7.691 (7.017,9.502)	7.823±1.229<br>7.808 (6.674,8.549)	7.155±1.391<br>7.627 (6.465,8.157)	7.526±1.26<br>6.688 (5.857,8.095)	7.405±1.731<br>7.979 (7.149,8.364)	7.457±1.72<br>7.665 (6.159,8.031)	20.63

C18:1n9t	Methyl elaidate	0.685±0.419<br>0.552 (0.313,0.838)	0.723±0.410<br>0.229 (0.18,0.584)	0.664±0.405<br>0.329 (0.247,0.53)	0.6±0.369<br>0.472 (0.306,0.827)	0.647±0.446<br>0.908 (0.793,1.191)	0.528±0.342<br>0.964 (0.663,1.178)	61.68
C18:1n9c	Cis-9-Octadecanoic acid	37.083±4.695<br>35.445 (33.706,38.914)	36.691±3.430<br>38.243 (35.77,42.813)	38.303±3.382<br>38.541 (35.029,39.708)	37.648±3.872<br>38.569 (34.175,43.212)	37.526±3.794<br>35.813 (33.696,37.309)	37.569±3.307<br>37.766 (35.532,39.741)	9.93
C18:1n7c	11-Octadecenoic acid	1.915±0.292<br>2.019 (1.396,2.204)	1.751±0.562<br>1.818 (1.663,1.952)	1.669±0.566<br>1.66 (1.473,2.021)	1.727±0.524<br>1.908 (1.767,2.049)	1.716±0.392<br>1.709 (1.239,1.997)	1.727±0.435<br>1.91 (1.648,2.096)	26.71
C18:2n6t	Methyl linolelaidate (trans-9, trans-12 octadecadienic acid)	0.073±0.049<br>0.043 (0.032,0.087)	0.070±0.050<br>0.010 (0.010,0.040)	0.069±0.043<br>0.034 (0.014,0.056)	0.068±0.045<br>0.061 (0.041,0.124)	0.078±0.053<br>0.115 (0.093,0.122)	0.061±0.039<br>0.116 (0.098,0.128)	65.76
C18:2	Cis-9, trans-12 octadecadienic acid	0.087±0.077<br>0.037 (0.024,0.076)	0.083±0.058<br>0.010 (0.010,0.023)	0.073±0.056<br>0.043 (0.012,0.056)	0.069±0.053<br>0.056 (0.042,0.114)	0.078±0.084<br>0.108 (0.091,0.133)	0.063±0.051<br>0.136 (0.11,0.164)	84.07
C20:0	Methyl arachidate	0.216±0.090<br>0.183 (0.13,0.261)	0.214±0.072<br>0.218 (0.184,0.312)	0.206±0.098<br>0.280 (0.253,0.303)	0.201±0.075<br>0.260 (0.204,0.289)	0.212±0.089<br>0.169 (0.156,0.182)	0.205±0.092<br>0.158 (0.134,0.179)	40.48
C18:2	Trans-9, cis-12 octadecadienic acid	0.099±0.124<br>0.146 (0.039,0.234)	0.096±0.117<br>0.010 (0.010,0.010)	0.066±0.068<br>0.010 (0.010,0.010)	0.086±0.08<br>0.017 (0.010,0.058)	0.072±0.092<br>0.200 (0.108,0.254)	0.063±0.102<br>0.025 (0.01,0.075)	121.69
C18:2	Methyl linoleate (cis-9, cis-12 octadecadienic acid)	10.852±2.993<br>9.019 (8.193,12.244)	11.666±2.573<br>11.584 (11.208,12.624)	11.801±3.291<br>11.252 (8.917,14.908)	11.804±2.401<br>14.275 (11.461,15.989)	11.488±3.38<br>10.728 (9.59,12.967)	11.757±2.971<br>9.946 (9.033,11.261)	25.13
C20:1n9	Cis-11-eicosenoic acid	0.433±0.131<br>0.468 (0.4,0.496)	0.419±0.096<br>0.378 (0.320,0.431)	0.411±0.095<br>0.373 (0.277,0.484)	0.374±0.136<br>0.490 (0.406,0.531)	0.393±0.107<br>0.341 (0.311,0.380)	0.387±0.088<br>0.404 (0.362,0.42)	27.23
C21:0	Methyl heneicosanoate	0.041±0.036<br>0.017 (0.011,0.032)	0.038±0.033<br>0.010 (0.010,0.010)	0.033±0.026<br>0.010 (0.010,0.027)	0.038±0.049<br>0.044 (0.030,0.075)	0.037±0.037<br>0.030 (0.024,0.034)	0.032±0.029<br>0.051 (0.035,0.067)	96.44
C18:3n6	Methyl y-linoleate	0.073±0.055<br>0.078 (0.057,0.085)	0.061±0.041<br>0.066 (0.016,0.114)	0.081±0.049<br>0.118 (0.049,0.135)	0.07±0.055<br>0.060 (0.035,0.084)	0.065±0.047<br>0.030 (0.010,0.047)	0.058±0.046<br>0.039 (0.033,0.061)	71.57
C18:2 unknown1		0.016±0.010<br>0.023 (0.018,0.04)	0.021±0.023<br>0.010 (0.010,0.015)	0.017±0.012<br>0.010 (0.010,0.026)	0.016±0.012<br>0.010 (0.010,0.010)	0.018±0.016<br>0.010 (0.010,0.010)	0.015±0.011<br>0.01 (0.01,0.01)	85.06
C18:2 unknown2		0.028±0.022<br>0.021 (0.012,0.031)	0.027±0.017<br>0.010 (0.010,0.010)	0.023±0.016<br>0.026 (0.010,0.043)	0.041±0.059<br>0.030 (0.016,0.045)	0.03±0.026<br>0.029 (0.026,0.046)	0.025±0.024<br>0.01 (0.01,0.023)	106.48
C22:0	Methyl docosanate	1.203±0.498<br>1.084 (0.856,1.298)	1.204±0.507<br>1.010 (0.870,1.342)	1.365±0.729<br>1.102 (0.964,1.402)	1.222±0.495<br>1.467 (0.97,1.633)	1.283±0.68<br>1.377 (0.980,1.756)	1.218±0.359<br>0.991 (0.726,1.256)	43.92
C18:3n3	Methyl linolenate	0.034±0.017<br>0.032 (0.013,0.050)	0.039±0.026<br>0.010 (0.010,0.021)	0.035±0.024<br>0.022 (0.010,0.045)	0.041±0.031<br>0.046 (0.038,0.070)	0.039±0.027<br>0.043 (0.029,0.051)	0.038±0.021<br>0.038 (0.028,0.048)	64.7
C20:2	Methyl cis,cis 11, 14 eisosadienoate (cis-11,14-eicosadienioc acid)	0.607±0.217<br>0.594 (0.429,0.68)	0.608±0.199<br>0.386 (0.301,0.857)	0.531±0.184<br>0.447 (0.341,0.514)	0.546±0.192<br>0.491 (0.382,0.706)	0.582±0.247<br>0.719 (0.629,0.787)	0.496±0.213<br>0.6 (0.524,0.737)	37.17
C22:1n9	Methyl erucate	0.044±0.027<br>0.029 (0.019,0.096)	0.043±0.032<br>0.010 (0.010,0.016)	0.044±0.033<br>0.025 (0.010,0.043)	0.048±0.034<br>0.064 (0.052,0.076)	0.043±0.033<br>0.054 (0.031,0.067)	0.034±0.028<br>0.036 (0.021,0.054)	72.71

C23:0	Methyl tricosanoate	0.010±0.001<br>0.010 (0.010,0.010)	0.010±0.000<br>0.010 (0.010,0.010)	0.010±0.001<br>0.010 (0.010,0.010)	0.015±0.023<br>0.010 (0.010,0.010)	0.02±0.041<br>0.010 (0.010,0.010)	0.015±0.02<br>0.01 (0.01,0.01)	153.01
C20:3n6	Cis-8,11,14-eicasatrienioc acid	0.341±0.185<br>0.343 (0.298,0.391)	0.269±0.142<br>0.206 (0.184,0.308)	0.261±0.113<br>0.264 (0.225,0.358)	0.242±0.110<br>0.289 (0.249,0.361)	0.216±0.111<br>0.274 (0.249,0.302)	0.19±0.12<br>0.01 (0.010,0.145)	54.54
C20:3n3	Cis-11,14,17-eicasatrienioc acid	0.045±0.077<br>0.024 (0.020,0.038)	0.022±0.037<br>0.010 (0.010,0.010)	0.022±0.033<br>0.010 (0.010,0.010)	0.026±0.056<br>0.010 (0.010,0.010)	0.034±0.059<br>0.010 (0.010,0.010)	0.04±0.085<br>0.08 (0.010,0.201)	190.65
C20:4	Methyl arachidonate	0.327±0.145<br>0.282 (0.249,0.312)	0.311±0.099<br>0.292 (0.236,0.317)	0.29±0.126<br>0.317 (0.260,0.394)	0.282±0.120<br>0.375 (0.339,0.447)	0.269±0.14<br>0.363 (0.304,0.396)	0.241±0.137<br>0.01 (0.010,0.252)	44.94
C24:0	Methyl tetracosanoate	0.015±0.014<br>0.010 (0.010,0.013)	0.016±0.011<br>0.010 (0.010,0.010)	0.012±0.004<br>0.010 (0.010,0.010)	0.013±0.009<br>0.010 (0.010,0.019)	0.014±0.010<br>0.010 (0.010,0.011)	0.013±0.006<br>0.01 (0.010,0.017)	68.46
C18:1cis6	6-octadecenoic acid	0.012±0.005<br>0.010 (0.010,0.010)	0.014±0.007<br>0.010 (0.010,0.010)	0.013±0.007<br>0.010 (0.010,0.010)	0.013±0.007<br>0.019 (0.010,0.027)	0.015±0.013<br>0.012 (0.010,0.019)	0.017±0.012<br>0.011 (0.010,0.017)	63.09
C22:2n6	Cis-13,16-docosadienoic acid	0.015±0.010<br>0.010 (0.010,0.011)	0.020±0.012<br>0.010 (0.010,0.010)	0.014±0.008<br>0.010 (0.010,0.010)	0.020±0.015<br>0.016 (0.010,0.027)	0.014±0.007<br>0.019 (0.011,0.026)	0.013±0.008<br>0.011 (0.010,0.025)	64.65
C18:2 unknown3		0.082±0.052<br>0.077 (0.065,0.115)	0.089±0.043<br>0.025 (0.010,0.097)	0.06±0.045<br>0.061 (0.040,0.071)	0.055±0.029<br>0.051 (0.014,0.088)	0.056±0.035<br>0.075 (0.049,0.093)	0.046±0.029<br>0.059 (0.035,0.067)	64.32
C24:1n9	Methyl cis-15-tetracosenoate	0.010±0.001<br>0.010 (0.010,0.019)	0.013±0.006<br>0.010 (0.010,0.010)	0.013±0.006<br>0.010 (0.010,0.012)	0.015±0.009<br>0.010 (0.010,0.011)	0.015±0.011<br>0.011 (0.010,0.013)	0.011±0.006<br>0.010 (0.010,0.011)	54.53
C18:1n9	9-octadecenoic acid	0.015±0.009<br>0.010 (0.010,0.010)	0.013±0.007<br>0.010 (0.010,0.010)	0.012±0.003<br>0.01 (0.010,0.010)	0.013±0.005<br>0.015 (0.010,0.020)	0.011±0.002<br>0.011 (0.010,0.014)	0.013±0.009<br>0.010 (0.010,0.013)	50.86
C20:5n3	Cis 5,8,11,14,17-eicosapentaenoic acid	0.085±0.112<br>0.035 (0.023,0.048)	0.065±0.051<br>0.032 (0.012,0.063)	0.065±0.054<br>0.07 (0.046,0.080)	0.061±0.048<br>0.091 (0.060,0.179)	0.061±0.056<br>0.056 (0.038,0.132)	0.052±0.048<br>0.012 (0.01,0.033)	99.83
C18:2 unknown4		0.036±0.033<br>0.024 (0.017,0.035)	0.024±0.019<br>0.010 (0.010,0.019)	0.030±0.023<br>0.022 (0.010,0.026)	0.029±0.029<br>0.011 (0.010,0.018)	0.024±0.021<br>0.016 (0.010,0.033)	0.024±0.02<br>0.029 (0.022,0.07)	87.06
C18:1-13E	Trans-13-octadecenoic acid	0.012±0.005<br>0.010 (0.010,0.010)	0.014±0.014<br>0.010 (0.010,0.010)	0.014±0.015<br>0.010 (0.010,0.010)	0.013±0.012<br>0.01 (0.010,0.010)	0.016±0.026<br>0.010 (0.010,0.014)	0.014±0.013<br>0.01 (0.01,0.01)	108.77
C22:4n3	Cis-7,10,13,16-docosatetraenoic acid	0.126±0.107<br>0.113 (0.094,0.121)	0.112±0.065<br>0.088 (0.037,0.147)	0.105±0.056<br>0.117 (0.08,0.143)	0.102±0.065<br>0.132 (0.068,0.202)	0.111±0.057<br>0.079 (0.023,0.124)	0.098±0.076<br>0.119 (0.022,0.142)	66.07
C22:6 n3	Methyl 4,7,10,13,16,19-docosahexaenoate	0.225±0.163<br>0.096 (0.077,0.152)	0.219±0.124 0.08 (0.010,0.148)	0.205±0.156<br>0.264 (0.209,0.325)	0.192±0.128<br>0.248 (0.133,0.472)	0.195±0.181<br>0.187 (0.149,0.360)	0.145±0.106<br>0.114 (0.101,0.15)	73.31

Supplementary Table S4. Significant relationships between human milk fatty acids and maternal intake frequency for eggs, nuts and avocado

Fatty acid	Food	p value
Hexanoic acid (C6:0)	Eggs	0.041
Decanoic acid (C10:0)	Eggs	0.02
Dodecanoic acid (C12:0)	Eggs	0.024
Arachidic acid (C20:0)	Eggs	0.005
Linoleic acid (C18:2n6)	Eggs	0.018
Erucic acid (C22:1)	Eggs	0.008
Eicasatrienoic acid (C20:3n6)	Eggs	<0.001
Conjugated linoleic acid unknown 2 C18:2)	Eggs	<0.001
Eicosapentaenoic acid (C20:5n3)	Eggs	0.001
Docosahexaenoic acid (C22:6n3)	Eggs	0.003
Dodecanoic acid (C12:0)	Nuts	0.008
Myristoleic acid (C14:1)	Nuts	0.016
Octadecanoic acid (C18:0)	Nuts	0.002
Heneicosanoic acid (C21:0)	Nuts	0.002
γ-linolenic acid (C18:3n6)	Nuts	<0.001
Decanoic acid (C10:0)	Avocado	0.008
Tridecanoic acid (C13:0)	Avocado	0.03
Tetradecanoic acid (C14:0)	Avocado	0.011
Palmitic acid (C16:0)	Avocado	0.042
Octadecanoic acid (C18:0)	Avocado	0.039
Linoleic acid (C18:2n6)	Avocado	<0.001
Arachidonic acid (C20:4)	Avocado	0.041
Eicosapentaenoic acid (C20:5n3)	Avocado	0.024
Docosahexaenoic acid (C22:6n3)	Avocado	0.002

Supplementary Table S5. Infant fatty acid intake (mg/day) for exclusively breastfeeding infants from months one to six of lactation. Results are presented as mean±SD.

Fatty acid	Month 1	Month 2	Month 3	Month 4	Month 5	Month 6
**Hexanoic acid (C6:0)**	3.05±1.08	3.43±1.94	3.05±0.96	5.21±2.31	3.87±2.28	4.75±3.48
**Octanoic acid (C8:0)**	36.11±34.26	63.88±48.03	58.39±58.54	43.62±18.11	33.98±24.42	30.32±28.65
**Decanoic acid (C10:0)**	433.51±171.64	531.9±222.7	607.39±315.83	488.55±207.61	406.15±148.84	441.22±164.43
**Undecanoic acid (C11:0)**	2.97±0.94	2.97±0.94	2.97±0.94	2.97±0.94	2.96±0.95	2.97±0.94
**Dodecanoic acid (C12:0)**	1436.39±610.92	1395.2±766.57	1484.69±888.82	1431.59±706.09	1189.34±459.23	1325.4±593.54
**Tridecanoic acid (C13:0)**	2.97±0.94	2.97±0.94	2.97±0.94	2.97±0.94	3.16±1.15	3.04±0.95
**Tetradecanoic acid (C14:0)**	1891.48±773.42	1509.21±732.67	1494.54±848.22	1426.64±756.66	1623.07±588.94	1755.43±778.38
**Pentadecanoic acid (C15:0)**	103.67±57.81	78.24±43.6	85.10±57.44	83.9±61.64	139.12±47.94	118.28±46.46
**Myristoleic acid (C14:1)**	62.97±44.49	52±29.93	56.40±46.72	70.5±70.49	95.02±34.29	91.35±38.2
**Palmitic acid (C16:0)**	6646.47±2013.92	6330.91±1980.67	6599.5±2216.91	5491.69±2138.67	6825.61±2342.27	6711.43±2091.36
**Cis-10-pentadecanoic acid (C15:1)**	2.97±0.94	2.97±0.94	2.97±0.94	2.97±0.94	2.97±0.94	2.97±0.94
**7-hexadecanoic acid (C16:1)**	145.25±101.61	102.16±48.09	93.88±42.32	143.26±59.52	153.91±44.2	150.08±51.56
**Heptadecanoic acid (C17:0)**	907.69±346.66	739.97±217.88	781.32±343.37	772.87±379.63	815.67±236.7	921.28±363.25
**Octadecanoic acid (C18:0)**	2439.46±929.96	2258.24±888.31	2104.78±589.01	2114.45±932.96	2286.16±1000.91	2110.78±639.81
**Elaidic acid (C18:1n9t)**	187.32±122.85	112.08±98.46	119.40±73.77	176.22±141.6	291.52±134.99	267.89±118.26
**Cis-9-octadecanoic acid (C18:1n9c)**	10744.83±3637.14	11486.18±3472.41	11185.02±3468.93	11373.13±3937.07	10748.1±3711.99	11162.07±4019.56
**11-octadecanoic acid (C18:1 n7c)**	492.22±247.34	523.2±180.3	514.03±180.5	547.79±197.67	449.34±235.14	565.43±224.76
**Trans-9, trans-12 octadecadienic acid (C18:2 n6t)**	19.26±16.87	6.96±6.71	10.93±8.31	22.23±15.49	31.77±10.23	33.18±11.85

**Cis-9, trans-12 octadecadienic acid (C18:2)**	15.58±12.75	5.27±4.42	12.26±9.75	27.5±22.62	32.75±15.47	43.39±24.27
**Arachidic acid (C20:0)**	60.22±42.57	73.43±48.59	76.55±22.3	75.77±40.62	49.73±18.88	44.21±13.96
**Trans-9, cis-12 octadecadienic acid (C18:2)**	49.34±39.18	7.97±15.10	2.97±0.94	12.85±15.81	58.16±34.04	17.23±23.22
**Cis-9, cis-12 octadecadienic acid linoleic (C18:2)**	2988.18±1135.6	3492.29±1058.68	3420.25±1331.11	4182.32±1577.55	3315.2±1244.81	3052.88±1093.21
**Cis-11-eicosenoic acid (C20:1n9)**	138.71±66.10	116.54±51.92	106.52±39.5	141.61±52.5	104.19±58.28	116.39±42.03
**Heneicosanoic acid (C21:0)**	9.98±14.65	3.78±2.1	5.56±3.47	16.56±9.48	11.78±11.05	16.08±8.44
**γ-linoleic acid (C18:3n6)**	24.67±16.41	21.44±18.43	29.58±18.45	21.44±18.04	11.16±10.82	14.16±8.31
**CLA unknown 1 (C18:2)**	8.80±5.54	5.57±6.61	6.62±5.82	3.68±2.2	2.97±0.94	3.05±0.91
**CLA unknown 2 (C18:2)**	10.81±17.73	6.23±9.5	8.86±7.43	10.2±7.79	11.67±9.76	5.63±4.42
**Docosanoic acid (C22:0)**	328.2±143.12	401.95±303.68	357.26±176.48	428.38±196.41	421.51±194.41	286.99±133.56
**Linolenic acid (C18:3n3)**	11.72±9.46	5.89±5.07	8.19±6.44	17.11±10.07	13.90±9.43	11.58±5.1
**Cis-11, cis-14 eisosadienoic acid (C20:2)**	175.8±86.04	166.06±119.01	129.87±57.24	156.03±79.63	202.37±62.42	177.95±70.63
**Tricosanoic acid (C23:0)**	16.11±14.91	4.90±4.36	10.04±8.73	19.45±10.55	16.72±11.52	12.75±8.91
**Cis-8,11,14-eicasatrienoic acid (C20:3n6)**	4.58±6.91	2.97±0.94	2.97±0.94	3.18±1.49	3.08±0.96	6.55±10.42
**Cis-11,14,17-eicasatrienoic acid (C20:3n3)**	108.14±52.55	78.32±50.81	80.94±42.22	92.86±63.85	79.74±29.58	28.41±41.1
**cis-11,14,17-eicasatrienioc acid ME C20:3N3**	9.59±6.99	3.65±2.26	2.95±0.95	3.84±3.47	2.97±0.94	34.97±41.97
**Arachidonic acid (C20:4)**	82.87±30.56	82.49±31.62	97.98±41.37	115.44±64.6	106.78±38.22	31.88±40.64
**Tetracosanoic acid (C24:0)**	4.80±4.72	2.97±0.94	3.56±1.75	4.41±2.56	4.22±3.31	4.12±2.07
**6-octadecanoic acid (C18:1cis6)**	3.20±1.53	2.97±0.94	3.04±0.84	6.70±5.28	4.88±4.03	4.51±2.42
**Cis-13,16-docosadienoic acid (C22:2n6)**	3.35±1.28	2.97±0.94	4.04±2.39	6.14±3.89	6.59±4.66	5.58±4.29

**CLA unknown 3 (C18:2)**	27.6±16.75	18.12±21.9	17.11±9.42	17.42±13.96	21.59±14.26	18.03±13.56
**Cis-15-tetracosanoic acid (C24:1n9)**	5.10±4.32	2.97±0.94	4.16±2.26	3.68±1.97	3.78±1.81	3.52±1.48
**9-octadecanoic acid (C18:1n9)**	3.27±1.51	2.97±0.94	3.19±1.12	5.42±4.27	3.98±2.28	4.46±3.35
**Cis-5,8,11,14,17-eicosapentanoic acid (C20:5n3)**	10.73±6.76	15.37±17.37	19.44±10.32	33.30±23.78	30.47±31.98	6.93±5.79
**CLA unknown 4 (C18:2)**	8.39±5.96	7.55±12.10	8.31±7.71	6.97±10.46	6.28±4.19	14.74±11.66
**Trans-13-octadecenoic acid (C18:1-13E)**	4.63±4.87	2.97±0.94	4.33±5.48	3.17±1.41	5.3±7.77	3.89±3.94
**Cis-7,10,13,16-docosatetraenoic acid (C22:6n3)**	34.62±17.51	31.29±27.77	34.78±20.58	37.1±21.3	27.85±30.95	29.08±24.77
**Docosahexaenoic acid (C22:6)**	38.53±30.49	33.23±36.98	76.8±34.18	82.72±57.6	73.41±49.54	40.73±24.22
**AA:DHA**	3.25±2.18	8.97±9.37	1.31±0.29	2.22±2.16	1.83±0.93	0.77±1.01
**Total *n*-6**	3.23±1.23	3.69±1.12	3.65±1.41	4.45±1.69	3.56±1.30	3.17±1.13
**Total *n*-3**	0.11±0.06	0.09±0.08	0.14±0.06	0.17±0.09	0.15±0.11	0.12±0.06
***n*-6:*n*-3 ratio**	40.11±32.31	84.50±83.29	26.79±7.33	30.42±16.89	30.02±12.05	31.54±22.92
**Total saturated**	14311.55±4676.92	13402.02±4719.64	13670.59±5030.53	12392.75±4505.37	13819.4±4551.55	13782.84±4287.39
**Total unsaturated**	15434.60±5056.83	16402.58±4924.77	16074.87±5026.45	17343.03±5791.15	15915.34±5346.62	15954.69±5492.28
**Total monounsaturated**	11806.60±3924.53	12411.91±3770.85	12102.99±3737.2	12493.9±4179.04	11879.71±4031.52	12385.3±4393.09
**Total polyunsaturated**	3628.00±1381.68	3990.67±1248.94	3971.88±1496.58	4849.14±1721.17	4035.63±1450.49	3569.39±1228.07
